# Assessment of AI-Based Protein Structure Prediction for the NLRP3 Target

**DOI:** 10.3390/molecules27185797

**Published:** 2022-09-07

**Authors:** Jian Yin, Junkun Lei, Jialin Yu, Weiren Cui, Alexander L. Satz, Yifan Zhou, Hua Feng, Jason Deng, Wenji Su, Letian Kuai

**Affiliations:** WuXi AppTec (Shanghai) Co., Ltd., 240 Hedan Road, Shanghai 200131, China

**Keywords:** AlphaFold, RoseTTAFold, protein structure prediction, molecular dynamics simulations, NLRP3, MCC950

## Abstract

The recent successes of AlphaFold and RoseTTAFold have demonstrated the value of AI methods in highly accurate protein structure prediction. Despite these advances, the role of these methods in the context of small-molecule drug discovery still needs to be thoroughly explored. In this study, we evaluated whether the AI-based models can reliably reproduce the three-dimensional structures of protein–ligand complexes. The structure we chose was NLRP3, a challenging protein target in terms of obtaining a three-dimensional model both experimentally and computationally. The conformation of the binding pockets generated by the AI models was carefully characterized and compared with experimental structures. Further molecular docking results indicated that AI-predicted protein structures combined with molecular dynamics simulations offers a promising approach in small-molecule drug discovery.

## 1. Introduction

High-resolution X-ray crystallographic or NMR structure predictions of proteins and protein–ligand complexes are critical for understanding the interactions between small-molecule drug candidates and their targets, and therefore, are pivotal in the drug discovery process [1]. However, due to the limitations of experimental techniques, a large portion of protein structures remain unsolvable. Studies on novel drug targets are often restrained by a lack of protein structures with sufficient resolution or completeness.

AI methods for protein structure predictions, including AlphaFold (AF) [2] and RoseTTAFold (RF) [3], have attracted tremendous interest for their ability to accurately predict protein structures, without relying on structural templates with high sequence similarity. Despite the recent success of these methods in predicting structures of single proteins and protein-protein complexes, their applications in small-molecule drug discovery have not been systematically investigated yet. One of barriers to using AI predicted structures is that protein targets may undergo induced-fit conformational changes upon the binding of small molecules [4]. An additional challenge for future research concerns how we can apply these methods to make structure predictions when the investigated protein comprises multiple domains or subdomains [5].

In the current study, we explored whether AI-predicted protein structures can be employed to determine the binding modes of known NLRP3 inhibitors. The target, NLRP3 (NOD-like receptor family, pyrin domain-containing protein 3) [6], is a protein linked to many chronic inflammatory human diseases, such as atherosclerosis, Alzheimer’s disease and nonalcoholic steatohepatitis. It is part of the NLRP3 inflammasome, which, when activated, triggers the release of proinflammatory cytokines IL-1β and IL-18, and thus leads to an inflammatory form of cell death [7]. Despite the considerable interest in this target, a lack of structural information has hindered the development of small-molecule inhibitors. We, therefore, carefully evaluated the quality of the AI-predicted models and assessed discrepancies between the binding pockets formed in the AI models and experimental structures. In this paper, we demonstrate that AI models combined with molecular dynamics (MD) simulations are valuable for exploring the mechanisms of small-molecule drug candidates and facilitating the lead optimization process.

## 2. Materials and Methods

### 2.1. Model Generation

Models generated by locally installed AlphaFold and RoseTTAFold were used as initial structures for subsequent docking and MD simulations; those models can be downloaded from the Supplementary Materials. Note that when querying templates for the protein target, we used the PDB downloaded on 14 May 2020 and the PDB70 cluster database downloaded on 13 May 2020, both before the release dates of the experimental structures 7PZC [8] and 7ALV [9].

### 2.2. Simulation Details

The docking poses were obtained using MOE [10]. All MD simulations were performed using the AMBER20 [11] program. Each system was solvated with TIP3P [12] water molecules in a rectangular box with a 10 Å buffer distance. For the small molecules, partial atomic charges were derived using AM1-BCC [13]. Bonded and Lennard-Jones parameters were obtained from GAFF2 [14]. The Amber force field ff19SB [15] was used for the protein. Counterions were only added to neutralize the total charge of each system.

The whole system was first minimized using a steepest descent algorithm, with the maximum number of cycles set as 10,000. Subsequently, the system was heated from 297 K to 300 K over a period of 50 ps. This was followed by 100 ps NVT and 200 ps NPT equilibrations, with all heavy atoms restrained during this process.

The production phase was simulated in an NPT ensemble with the Berendsen barostat [16] and Langevin [17] thermostat at 300 K without restraints. The cutoff distance of Lennard-Jones interactions was set to 10 Å and a long-range correction was applied to approximate the interactions beyond the cutoff distance. Electrostatic interactions were treated with the particle mesh Ewald (PME) scheme [18]. The simulation time of the production run was 200 nanoseconds (ns) for both the ADP-bound protein and the subsequent MCC950-docked complex.

## 3. Results

### 3.1. Evaluation of the AI-Predicted Models of NLRP3

**Figure 1:**
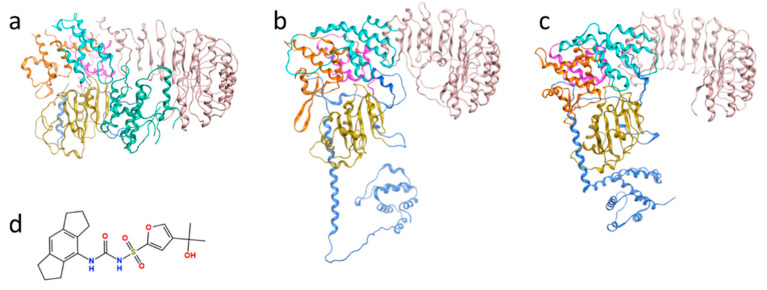
(**a**) Previously reported cryo-EM structures of NLRP3 complexed with NEK7 kinase (PDB code: 6NPY); the structures of NLRP3 predicted by (**b**) AlphaFold and (**c**) RoseTTAFold. Gold: NBD; Purple: HD1; Green: NEK7; Orange: WHD; Cyan: HD2; Pink: LRR; Blue: others. (**d**) The 2D structure of MCC950, which was reported as a selective NLRP3 inhibitor.

The human NLRP3 protein contains subunits PYD (pyrin domain; residues 3–95), FISNA (Fish-specific NACHT-associated domain; 96–218), LRR (leucine-rich repeat domain; 652–1036) and a central NACHT domain comprising NBD (nucleotide-binding domain; 219–372), HD1 (helical domain 1; 373–434), WHD (winged helix domain; 435–541) and HD2 (helical domain 2; 542–651) (Figure 1). For a long time, the only available experimental structure containing the central subdomains of this protein was a cryo-electron microscopy (cryo-EM) model bound to NEK7, a mitotic kinase-mediating NLRP3 activator (PDB code: 6NPY; Figure 1a). Yet, a substantial portion of the residues (31.8%) in the HD2 region near the small-molecule binding site in this structure was missing. In addition, the conformation of NLRP3 complexed with the NEK7 substrate may differ from the one bound to a small-molecule inhibitor. A cryo-EM structure of an NLRP3 decamer bound to the drug MCC950 (PDB code: 7PZC) was newly released on 26 January 2022 [8], and a crystal structure with an MCC950-like inhibitor (PDB code: 7ALV) was released on 20 October 2021 [9]. However, those two structures had not yet been deposited to the PDB when structural predictions were performed for this study.

Given the difficulties in obtaining the structure of the multi-subdomain NLRP3 protein using experimental and traditional theoretical methods, we chose this protein to test the accuracy of the AF and RF models when predicting protein conformations, and whether correct protein–small-molecule binding modes could be generated based on these structures. Five models were generated by two locally installed programs each. The pLDDT (predicted Local Distance Difference Test) scores [2] were calculated for every overall structure and subdomain to quantify the confidence in structure prediction. The scores were interpreted as follows: >90: high accuracy; 70–90: moderate to good; 50–70: low confidence; <50: disordered or unstructured. It was observed that the AF and RF models showed comparable overall confidence levels, with an average value of 79.9 and a standard deviation of 0.9 for AlphaFold, compared to 78.3 ± 1.2 for RoseTTAFold. It is also noteworthy that all pLDDT scores for the central subdomains were above 70, indicating that the protein was reliably predicted at the individual domain level.

For multi-domain or -subdomain protein targets such as NLRP3, the predicted Templated Modeling (pTM) score [2] can be used to measure the accuracy of domain packing. The AF package allows us to obtain pTM scores from finetuned pTM models (Table 1). In this study, numbers around 0.7 indicated moderate risk, associated with incorrect domain assembly and inter-domain contacts, which was of particular concern because the binding site of the known inhibitor MCC950 is located at the interfaces of several central subdomains.

Next, we computed the RMSD values between the predicted models and one of the monomers in the experimental NLRP3 decamer (PDB code: 7PZC). As shown in Table 2, the RF model performed better on WHD, while the prediction of the AF model in the HD2 region was closer to the experimental structure. In addition, when using both AF and RF methods, the RMSD values of the LRR subdomain structures were significantly higher than those of other subdomains, resulting in an overall RMSD value above 10 Å for all models. Focusing on subdomains and residues that play critical roles in small-molecule binding, we then performed a detailed analysis of the differences between the AI models and the experimental structures at the MCC950 binding sites. The results are described in the following section.

### 3.2. Characterization of the Binding Pocket of Compound MCC950

**Figure 2:**
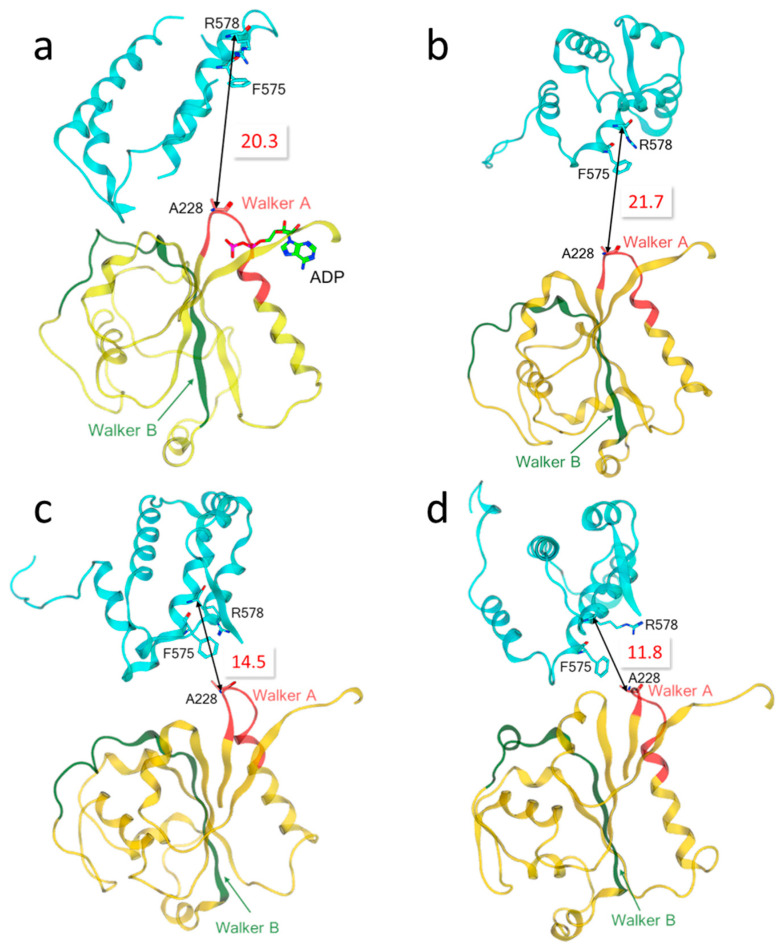
Relative positions between two key residues R578 and A228 of the MCC950 binding pocket in (**a**) the NLRP3-NEK7 complex structure (PDB code: 6NPY), (**b**) model 1 generated by AlphaFold, (**c**) model 4 by AlphaFold and (**d**) model 1 by RoseTTAFold. The distance between the α-carbon atoms of R578 and A228 was 20.3 Å, 21.7 Å, 14.5 Å and 11.8 Å in (**a**–**d**), respectively. Red: the Walker A motif (GAAGIGKTIL); Green: the Walker B motif (RILFMDGFDELQGAFDEHI) [19]; Gold: NBD; Cyan: HD2. Other domains were omitted for clarity.

**Figure 3:**
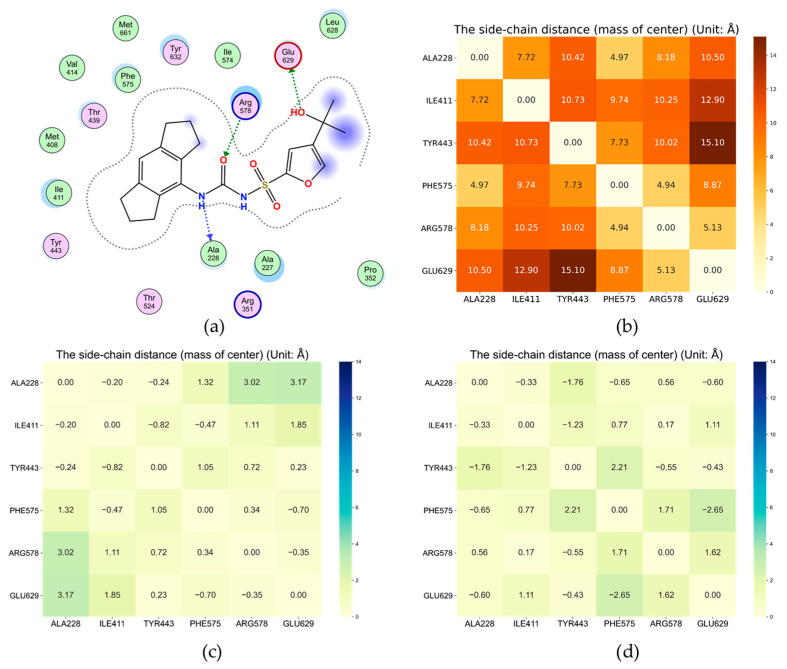
(**a**) Major interactions between MCC950 and NLRP3 in 7PZC; (**b**) side-chain distances between pairs of key residues at the binding site of MCC950 in 7PZC; side-chain distances between pairs of key residues in (**c**) AF model 4 and (**d**) RF model 1 relative to those in 7PZC (unit: Å). The magnitudes of the numbers in (**c**,**d**) indicate deviations from the experimental structure, and the plus and minus signs indicate larger or smaller distances compared to those in 7PZC.

**Figure 4:**
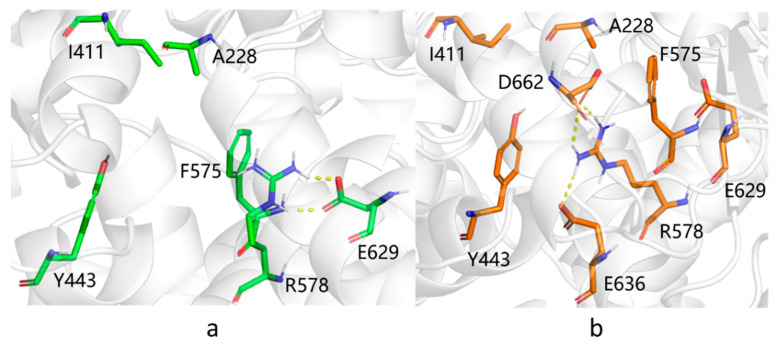
Salt bridges in the MCC950 binding pocket of (**a**) AF model 4 and (**b**) RF model 1.

MCC950 has long been identified as a selective NLRP3 inhibitor that blocks NLRP3 inflammasome activation [19,20] (Figure 1d), yet the underlying mechanism was unclear until very recently. The newly reported experimental structure showed that MCC950 is bound to a cleft formed by subdomains NBD, HD1, WHD, HD2 and LRR [8], which is separated from the ADP binding site by the Walker A motif (GAAGIGKTIL) [21]. The residues adjacent to MCC950 include F575, R578 and E629 in the HD2 subdomain, A228 in the Walker A motif of NBD as well as I411 and Y443 in HD1 (Figure 2 and Figure 3a). Therefore, we measured the distances among the α-carbon atoms of these key residues in each AI model, as well as the distances among their side chains, to assess the possibility of MCC950 binding pocket formation. In particular, we found that the carbamide and sulfonyl groups of MCC950 engaged hydrogen bonds with residues A228 and R578, found in different subdomains across the binding pocket (Figure 4a). This led us to consider the distance between A228 and R578 residues as one of the key indicators for the formation of a suitable binding site.

The results showed that R578 and its adjacent residue F575 in the experimental structure of the NLRP3-NEK7 complex (PDB code: 6NPY) were located in the solvent-exposed region, relatively distant from the ADP-binding site (Figure 2a). The distance between R578 and A228 was measured as 20.3 Å. In the AF models, the R578 and A228 residues faced each other, but the distance between them was around 20 Å in each AF structure, except for model 4 with a value of 14.5 Å, which indicated that the HD2 and NBD subdomains were not sufficiently packed together (Figure 2b,c) considering the corresponding distance of 12.7 Å in the experimental structure 7PZC. Note that the RMSD value between AF model 4 and the experimental structure was computed as the smallest value among all AI models (Table 2). In contrast, the HD2 and NBD subdomains in the RF models were more densely packed, and specifically, residues R578 and A228 residues at the interfaces seemed close enough (~12 Å) to simultaneously interact with the small-molecule inhibitor (Figure 2d).

In addition to the hydrogen bonds formed between the amide group of MCC950 and the protein, the tricyclic head group of the small molecule also participated in hydrophobic interactions with neighboring residues involving I411, Y443 and F575. In addition, the terminal carboxylate group engaged H-bonds with G629 (Figure 4a). We, therefore, compared the pairwise distances of related residues for all AF and RF models with those in 7PZC, to obtain a detailed measurement of binding pocket structural integrity. It was observed that, on average, AF model 4 had the smallest discrepancy from the experimental results among all AI models (Figure 3). Yet, the green areas shown in Figure 3c suggested a loosely packed core. In particular, a salt bridge between R578 and E629 was observed in the AF models, same as in 7PZC (Figure 4a), which further stabilized the complexation between the small molecule and the protein, whereas, in the RF models, R578 instead formed salt bridges with two other negatively charged residues, E636 and D662 (Figure 4b).

### 3.3. Refinement of the Docking Pose Using Molecular Dynamics Simulation

**Figure 5:**
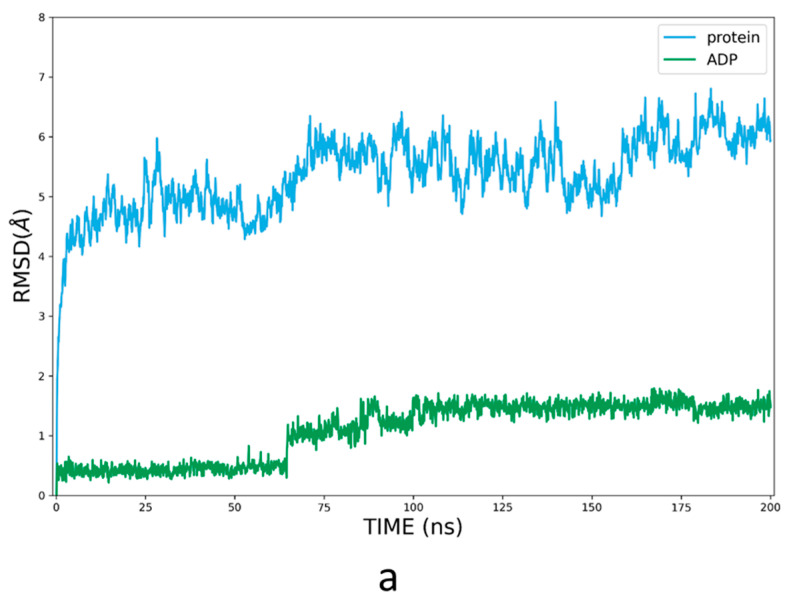

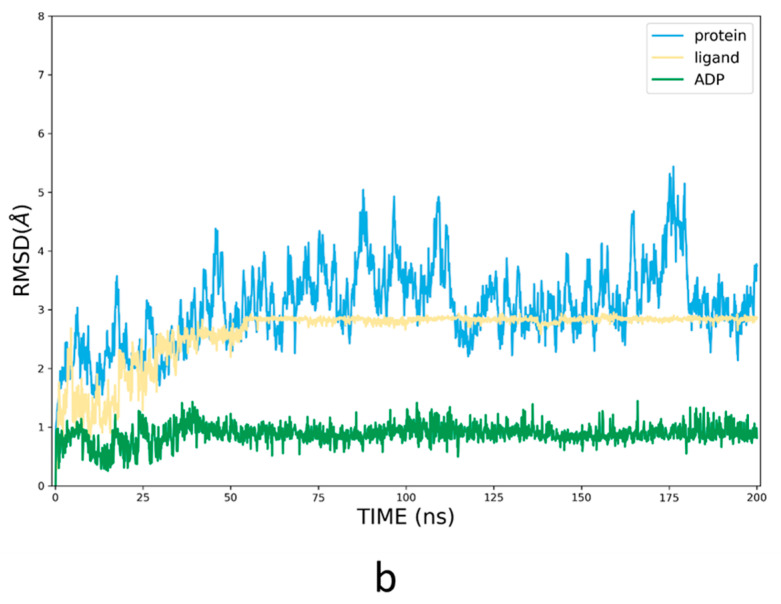
RMSD values plotted relative to the first frame (in Å) of the production phase based on the MD trajectories of (**a**) AF model 4 bound to ADP and (**b**) AF model 4 bound to ADP and MCC950. Blue line: Cα RMSDs of the protein; green and yellow lines: heavy-atom RMSDs of ADP and the ligand, respectively.

To further assess the use of AI models in small-molecule drug discovery, we performed molecular docking of MCC950 for each model of AF and RF. However, even the top-ranked models produced only partially correct binding modes. So, molecular dynamics (MD) simulations were carried out to refine the poses by considering the induced fit and protein dynamic effects. To keep the Walker A moiety from shifting, ADP was docked back into its binding site, as in the AI models, then simulated by MD for 200 ns. It turned out the RF model 1 structure could hardly accommodate ADP at the corresponding site. So, we only performed a 200 ns MD simulation of AF model 4 bound to ADP (Figure 5a). Another difficulty was that part of the FISNA domain missing from the experimental structure in AF Model 4 occupied the entrance of the binding pocket. Therefore, ligand MCC950 was docked to the protein–ADP conformation from the last frame of the simulation with the PYD and FISNA subdomains truncated, then simulated for another 200 ns (Figure 5b).

**Figure 6:**
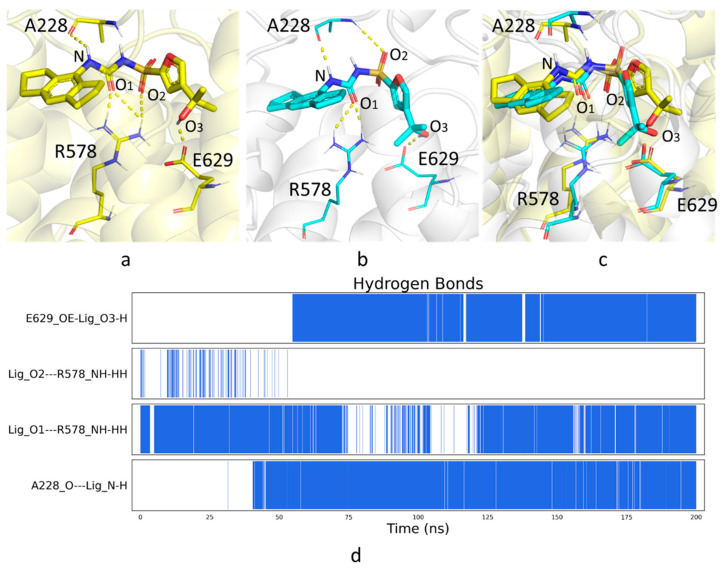
Binding modes of MCC950 in (**a**) 7PZC and (**b**) AF model 4 in complex with ADP and MCC950 from the last frame of the 200 ns MD simulations; (**c**) structural overlay of (**a**,**b**); (**d**) hydrogen bond analysis of the MD simulations.

Compared to the experimental structure, ligand MCC950 engaged similar hydrogen-bonding interactions with R578, A228 and G629 (Figure 6). The distance between the α-carbon atoms of R578 and A228 was reduced to 14.1 Å from the initial 14.5 Å, moving closer to the measured value of 12.7 Å in 7PZC. It was challenging to fully optimize the assembly of subdomains through MD simulations, as conformational changes involving the main chain typically require a long time to converge.

## 4. Discussion

Despite the use of protein structure templates during both training and prediction procedures, AlphaFold and RoseTTAFold are intrinsically more advanced than the conventional template-based methods such as homology modeling and threading. The neural network architecture enables AI methods to learn deeply from the coevolution of protein sequences, and detect the underlying correlations between pairs of amino acids through the construction of multiple sequence alignment (MSA) and pair representation, instead of merely patching the templates together. Therefore, the AI methods can accurately predict protein structures using templates with a sequence identity and structural coverage below 30%. This study demonstrated that the structures predicted by the AI methods did not heavily rely on the existing experimental structure, meaning they could be useful complements for experimental approaches including X-ray crystallography, NMR and cryo-EM.

AlphaFold has been shown to predict protein backbone as well as side chain coordinates with high accuracy. However, accurately predicting the protein structure was particularly challenging in the context of this work, as the NLRP3 protein consists of multiple subdomains, and the small-molecule binding pocket is located in the interdomain region. It was shown that both AlphaFold and RoseTTAFold can predict confidently at the subdomain level. Nevertheless, the subdomains were more compact in RoseTTAFold models in the present study, whereas AlphaFold successfully reproduced a key salt bridge interaction observed in the experimental structure. Our study highlights why AI protein structure prediction tools should be used with caution for drug targets comprising two or more structural domains, especially when the drug binding site is located at the interfaces of subunits. To help overcome difficulties, MD simulations can be applied to finetune the details of models in complex systems. In the future, we will evaluate the performance of AlphaFold in small-molecule drug discovery when applied to further drug target systems.

## Figures and Tables

**Table 1:** Predicted Local Distance Difference Test (pLDDT) and predicted Templated Modeling (pTM) scores for the NLRP3 protein structures generated using AphaFold and RoseTTAFold.

Domain	Model 1	Model 2	Model 3	Model 4	Model 5
	AlphaFold				
NBD	83.3	85.3	86.8	83.5	85.7
HD1	82.8	84.9	85.4	82.1	84.3
WHD	70.2	71.5	74.2	71.0	71.2
HD2	76.8	78.6	79.4	77.2	79.5
LRR	84.6	85.0	86.4	85.3	84.7
Whole	79.1	80.3	81.4	79.1	79.8
	RoseTTAFold				
NBD	83.4	81.6	82.5	80.7	79.5
HD1	83.5	82.3	81.3	80.7	80.3
WHD	80.0	78.4	76.4	78.7	77.3
HD2	75.2	73.1	70.2	73.0	71.1
LRR	82.3	82.9	83.1	81.9	81.8
Whole	79.7	79.4	77.9	77.6	76.8

LDDT scores are reported on a 0–100 scale. Values higher than 90: high accuracy; 70–90: moderate to good; 50–70: low confidence; <50: disordered or unstructured. pTM scores (numbers in the parentheses) are on a 0–1 scale. Numbers higher than 0.7 indicate high accuracy.

**Table 2:** RMSD values between the AI-predicted NLRP3 structures and one of the monomers in 7PZC (unit: Å).

Domain	Model 1	Model 2	Model 3	Model 4	Model 5	No. of Aligned Atoms
AlphaFold						
NBD	4.4 (3.7)	4.4 (3.7)	4.4 (3.7)	4.4 (3.7)	4.4 (3.7)	1178 (154)
HD1	2.7 (1.8)	2.6 (1.7)	3.0 (2.2)	2.6 (1.6)	2.7 (1.8)	505 (62)
WHD	6.0 (5.3)	6.2 (5.5)	6.1 (5.4)	5.9 (5.2)	6.1 (5.4)	780 (98)
HD2	18.5 (18.3)	18.6 (18.4)	18.5 (18.3)	18.5 (18.3)	18.4 (18.3)	587 (75)
LRR	12.1 (11.8)	12.5 (12.1)	13.5 (13.1)	11.4 (11.2)	12.8 (12.5)	2713 (359)
Full-length	14.7 (14.4)	14.8 (14.4)	14.6 (14.2)	10.5 (10.2)	14.6 (14.2)	6192 (798)
	RoseTTAFold					
NBD	4.2 (3.5)	4.3 (3.6)	4.3 (3.6)	4.2 (3.6)	4.1 (3.5)	Same as AlphaFold
HD1	2.4 (1.4)	2.6 (1.5)	2.5 (1.5)	2.5 (1.5)	2.5 (1.4)	
WHD	3.2 (2.3)	3.2 (2.3)	3.3 (2.4)	3.2 (2.3)	3.2 (2.4)	
HD2	16.3 (15.8)	17.6 (17.2)	17.6 (17.6)	16.4 (16.0)	16.5 (16.0)	
LRR	7.9 (7.4)	8.2 (7.7)	8.2 (7.7)	8.2 (7.7)	8.1 (7.5)	
Full-length	10.7 (10.2)	10.5 (9.9)	10.7 (10.2)	10.5 (9.9)	10.5 (9.9)	

Numbers before and in the parentheses are the RMSD values calculated using coordinates of all heavy atoms and α-carbons, respectively. Note that the RMSD values of the subunits were computed by superposing each set of predicted and experimental subdomains separately.

## Data Availability

Not applicable.

## Supplementary Materials

The following supporting information can be downloaded at: https://www.mdpi.com/article/10.3390/molecules27185797/s1, Raw models generated by AlphaFold and RoseTTAFold.
